# Studies of the Thermophysical Properties of 42CrMo4 Steel Manufactured Conventionally and via Laser Powder Bed Fusion (L-PBF) †

**DOI:** 10.3390/ma19061070

**Published:** 2026-03-11

**Authors:** Piotr Koniorczyk, Mateusz Zieliński, Janusz Zmywaczyk, Bartłomiej Sarzyński

**Affiliations:** 1 Faculty of Mechatronics, Armament and Aerospace, Military University of Technology, ul. gen. S. Kaliskiego 2, 00-908 Warsaw, Poland; mateusz.zielinski@wat.edu.pl (M.Z.); janusz.zmywaczyk@wat.edu.pl (J.Z.); 2 Faculty of Mechanical Engineering, Military University of Technology, ul. gen. S. Kaliskiego 2, 00-908 Warsaw, Poland; bartlomiej.sarzynski@wat.edu.pl

**Keywords:** additive manufacturing, 40HM steel (42CrMo4, AISI 4140 steel, 1.7225), laser-powder bed fusion, thermophysical properties, thermal diffusivity, specific heat capacity, thermal expansion, build orientation

## Abstract

In this work, measurements of thermal diffusivity, heat capacity and thermal expansion of 40HM (42CrMo4, 1.7225, AISI 4140) steel manufactured conventionally and via Laser Powder Bed Fusion (L-PBF) were carried out in the temperature range from room temperature (RT) to 1000 °C. Thermophysical properties were tested using specialized test stands from NETZSCH. Thermal diffusivity was studied using both the LFA 427 laser flash apparatus and the LFA 467 xenon flash apparatus. Specific heat capacity was investigated using DSC 404 F1 Pegasus differential scanning calorimeter, and thermal expansion was investigated using the DIL 402 C. Inconel 600 and A310 steel were selected as the reference materials during the thermal diffusivity test using LFA467 in the RT÷500 °C range. The conventionally manufactured 40HM steel, in the form of hot-rolled bar stock, was subjected to standard heat treatment for this steel grade—quenching followed by high-temperature tempering. The additively manufactured 40HM steel was subjected to stress-relief annealing. The results revealed no significant differences between the thermophysical properties of the L-PBF-produced samples in the out-of-plane and in-plane build orientations. Furthermore, no substantial differences were observed between the thermophysical properties of the conventionally produced material and the material manufactured using the L-PBF technique.

## 1. Introduction

Additive manufacturing (AM) techniques have become one of the fundamental methods for producing various components and machine parts [1,2,3,4]. Initially, the concept of AM was closely associated with the framework of the Industry 4.0 revolution; however, it can now also be integrated into the Industry 5.0 paradigm as a tool enabling sustainable and customized production [5,6]. The growing interest in AM technologies is evidenced by the increasing number of scientific publications addressing this subject [7,8,9]. According to ISO/ASTM 52900:2021, additive manufacturing is defined as “a process of joining materials to make parts from 3D model data, usually layer by layer” [10]. AM technologies are regarded as a complement to traditional manufacturing methods such as casting, forging, and machining [11]. Compared to these conventional methods, AM offers several key advantages, including exceptional design freedom (e.g., cellular structures, topology optimization), material efficiency (near-net-shaping), and the ability to fabricate integrated parts that would be difficult or impossible to produce using traditional techniques [12,13,14]. For example, by selecting various types of nanoparticles and controlling their dispersion in a metal matrix using powder metallurgy, engineers can obtain composites with specific tribological properties [15]. These benefits also contribute to reducing the overall carbon footprint of production processes [16].

Among all AM techniques, the most widespread are polymer-based methods such as FFF, FDM, or MEX, which utilize thermoplastic or composite materials [17]. These processes do not require advanced energy sources, such as laser beams or inert gas atmospheres, which make them accessible and cost-effective. When metallic materials are considered, the most applied methods include Powder Bed Fusion (PBF), Directed Energy Deposition (DED), Binder Jetting (BJ), Material Extrusion (ME), and Sheet Lamination [18]. Among these, PBF and DED techniques are the most widely used in metal additive manufacturing [19]. DED methods involve feeding the material directly into the melt pool, where it is fused using a focused energy source such as a laser (LP-DED/LMD), electron beam (EB-DED), plasma arc (PAD), or traditional electric arc (WAAM) [20]. In contrast, PBF techniques are based on the selective melting of thin layers of powder within a powder bed, using either a laser beam (LB-PBF/L-PBF) or an electron beam (EB-PBF) [21]. When comparing these two methods, L-PBF has clear advantages in the production of high-precision parts, primarily due to its ability to melt thin layers of material—typically ranging from 20 to 60 micrometers [22]. Numerous commercial L-PBF systems are available on the market, often integrated with in-process monitoring and quality-control solutions.

In metal AM, commonly used materials include aluminum alloys, titanium alloys, nickel-based superalloys, copper alloys, and various steels [23]. Among the most frequently employed steel grades are 316L, 17-4PH, maraging steels (e.g., M300), H13, H11, and duplex steels [24]. However, 40HM steel (also known as AISI 4140 or 42CrMo4) remains relatively underexplored in the context of L-PBF processing [25]. 42CrMo4 is a medium-carbon alloy steel intended for heat treatment, widely used in the manufacture of heavily loaded machine components such as shafts, connecting rods, and gears [26]. Conventionally produced parts made from this material are typically supplied in a quenched and tempered condition, owing to its excellent fatigue strength. In AM processing, components made from 40HM using the L-PBF method are subjected to stress-relief annealing, aimed at reducing residual stress generated during fabrication due to steep thermal gradients and rapid solidification of the melt pool.

In metal fabrication by AM, a separate issue is the search for correlations between thermophysical properties, microstructural characteristics, and mechanical behavior of steels subjected to various heat treatments [27]. For example, to study the effect of annealing on MgAZ31-H24, point pulsed laser thermography is used, focusing on the detection of microstructural changes through through-thickness thermal diffusivity measurements [28]. Laser infrared photothermal radiometric phase imaging is used to image the surface hardness profiles of heat-hardened steels [29]. Photoacoustic and photothermal methods offer a unique combination of features that make them very attractive for use in a wide range of scientific and technical applications, including the assessment of the properties of heterogeneous materials, including, for example, the characterization of thin steel layers [30].

**Figure 1:**
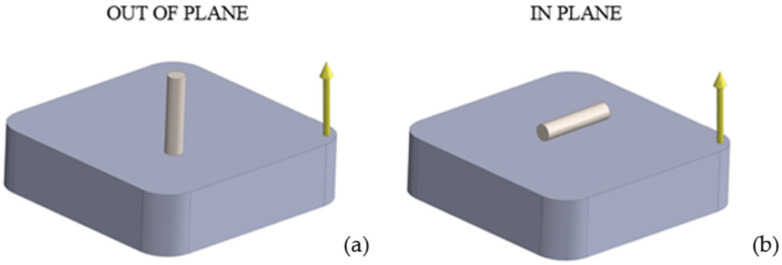
Schematic diagram of the sample orientation relative to the build platform: (**a**) out-of-plane, (**b**) in-plane. The arrow indicates the direction of material deposition and melting of subsequent layers during the L-PBF process [31].

The investigation of the thermophysical properties—specifically thermal diffusivity, heat capacity, and thermal expansion—of 40HM steel is essential for understanding its behavior under thermal loading. This understanding has direct implications for modeling wear, thermal fatigue, and heat-treatment processes, such as phase transformations. However, comprehensive data on the thermophysical properties of steels manufactured by additive techniques are lacking in the scientific literature. This study aims to compare these properties between conventionally produced and laser powder bed fusion (L-PBF) fabricated 40HM steel. The investigated materials were subjected to heat treatments characteristic of their respective manufacturing routes, i.e., the conventionally produced material was quenched and high-temperature tempered, whereas the L-PBF-manufactured material was subjected to stress-relief annealing. Two build orientations were analyzed for the L-PBF specimens: out-of-plane (perpendicular to the build platform) and in-plane (parallel to the build platform), as shown in Figure 1. Consequently, three sample sets were prepared: one from the conventional material and two from the additively manufactured variants. It should be emphasized that the present study focuses on bulk-averaged thermophysical properties determined using macroscopic measurement techniques (LFA, DSC, DIL). The objective is not to assess local sensitivity to microstructural heterogeneities (e.g., melt-pool morphology, defect distribution, or crystallographic texture), but rather to evaluate effective thermophysical parameters representative of the material volume at the measurement scale. The presented results are discussed in relation to the Curie temperature and shrinkage effects in L-PBF-produced 40HM steel. Furthermore, the data generated in this study serve as essential input parameters for numerical simulations of heat transfer in components made from this steel and operating within a wide temperature range, from RT to 1000 °C.

## 2. Material and Method

### 2.1. Material

The study employed 40HM steel (AISI 4140, 42CrMo4, 1.7225). Samples were produced from gas-atomized steel powder with a particle size of 20–53 μm and from a conventional hot-rolled rod. The powder was supplied by Höganäs (Ath, Belgium). The reference sample was machined from a hot-rolled rod supplied by ArcelorMittal (Warsaw, Poland) along its longitudinal axis. The chemical composition of both materials, as certified by the manufacturers, is provided in Table 1 [32,33].

### 2.2. Sample Preparation

**Figure 2:**
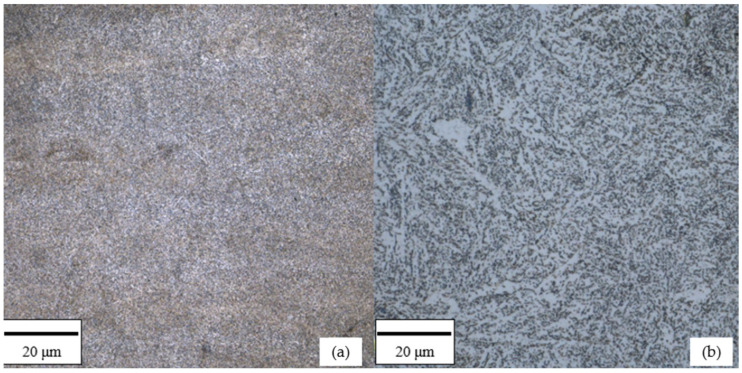
Optical microstructures of 42CrMo4 steel observed using a confocal optical microscope: (**a**) L-PBF-manufactured material after stress-relief annealing; (**b**) conventionally produced material after quenching and high-temperature tempering.

Samples manufactured using the Laser Powder Bed Fusion (L-PBF) technique were produced on an SLM 125 HL system (Nikon SLM Solutions, Lübeck, Germany). The fabrication process was carried out using a laser power of 302.5 W, a scanning speed of 810 mm/s, and a hatch spacing of 0.108 mm. The layer thickness was set to 0.03 mm. A scanning strategy based on parallel scan lines was applied, with three contour scans performed along the outer perimeter of each layer. Throughout the entire build process, the build platform was maintained at a temperature of 180 °C. Processing was conducted under an argon atmosphere, with the oxygen content kept below 0.1%. The material was fabricated using virgin powder obtained from a newly opened container supplied by the powder manufacturer. The porosity of the additively manufactured (L-PBF) samples was experimentally evaluated using two independent methods: metallographic analysis and X-ray computed tomography (CT). For the metallographic assessment, a representative L-PBF specimen was sectioned in two orthogonal planes corresponding to the out-of-plane (OOP) and in-plane (IP) build directions. Quantitative image analysis of polished cross-sections revealed an average porosity of 0.3%. The same specimen was subsequently examined using X-ray computed tomography (Waygate Technologies, Wunstorf, Germany), which enabled volumetric porosity evaluation and yielded a total porosity of 1.07%. The difference between the values obtained by metallography and CT is attributed to the limited two-dimensional nature of metallographic sections compared to the three-dimensional, volume-averaged character of CT measurements. The material produced via the L-PBF technique was subjected to stress-relief annealing to reduce residual stresses inherent to the additive manufacturing process. This treatment involved heating the samples in a furnace at 650 °C for two hours, followed by furnace cooling. The conventional material (Sample W) underwent a standard quenching and tempering cycle: quenching from 840 °C (held for 40 min) in oil, followed by high-temperature tempering at 650 °C for 2.5 h with subsequent air cooling. Figure 2 presents optical micrographs of the microstructure of 42CrMo4 steel manufactured by L-PBF after stress-relief annealing (Figure 2a) and conventionally produced steel after quenching and high-temperature tempering (Figure 2b). The observations were performed using a confocal optical microscope (Olympus Corporation, Tokyo, Japan).

The microstructure of the L-PBF-manufactured steel after stress-relief annealing (Figure 2a) is characterized by a relatively fine and homogeneous morphology, resulting from rapid solidification during the additive manufacturing process. The original melt-pool boundaries are not distinctly visible at the applied magnification, indicating effective stress-relief annealing and partial microstructural homogenization. The structure can be described as fine-grained, with features typical of additively manufactured steels subjected to subcritical heat treatment, where residual stresses are reduced without inducing full phase transformation. In contrast, the conventionally produced 42CrMo4 steel subjected to quenching and high-temperature tempering exhibits a microstructure characteristic of tempered martensite (Figure 2b). The structure consists of uniformly distributed carbide precipitates within a ferritic matrix, forming a characteristic lath-like morphology typical for quenched and tempered medium-carbon alloy steels. This microstructure reflects the equilibrium state achieved during high-temperature tempering, resulting in reduced internal stresses and stable mechanical and thermophysical properties. The observed differences in microstructural morphology are consistent with the distinct manufacturing routes and heat-treatment conditions applied to the investigated materials and should be taken into account when interpreting the measured thermophysical properties. These qualitative observations confirm that the compared materials differ in microstructural state, and this distinction must be considered when interpreting similarities or differences in bulk thermophysical properties.

All test specimens were prepared using electrical discharge machining (EDM; AccuteX Technologies, Taichung, Taiwan) to ensure the material structure remained unaltered. For the L-PBF material, samples were extracted in two orientations relative to the build platform: perpendicular (Out-Of-Plane, OOP) and parallel (In-Plane, IP), as illustrated in Figure 1. The specimens for thermophysical characterization—specifically thermal diffusivity (LFA), specific heat (DSC), and thermal expansion (DIL)—were prepared as disk-shaped samples (see Table 2). The density of the material measured at RT by double weighing (in air and water) using the SARTORIUS MSA125P-1CE-DA analytical balance (readability [d]: 0.01 mg; Sartorius Lab Instruments GmbH, Göttingen, Germany). The density ρ of the W sample was equal to 7.80 g·cm^−3^, while for the L-PBF samples (OOP and IP), it was 7.77 g·cm^−3^. Density measurements were performed using W and L-PBF thermal diffusivity test samples.

## 3. Results and Discussion

### 3.1. Thermal Diffusivity Results

**Figure 3:**
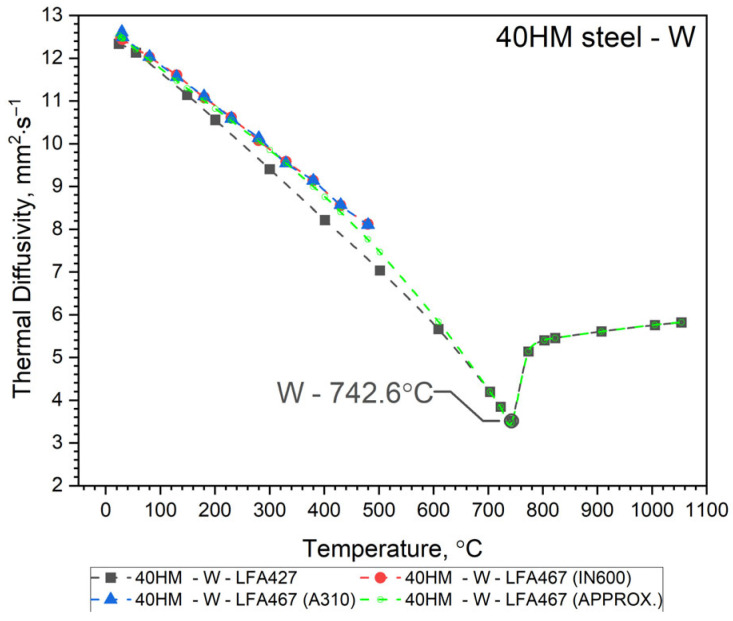
Thermal diffusivity as a function of temperature for 40HM (42CrMo4) steel obtained from LFA 467 and LFA 427 for sample W—conventional material.

**Figure 4:**
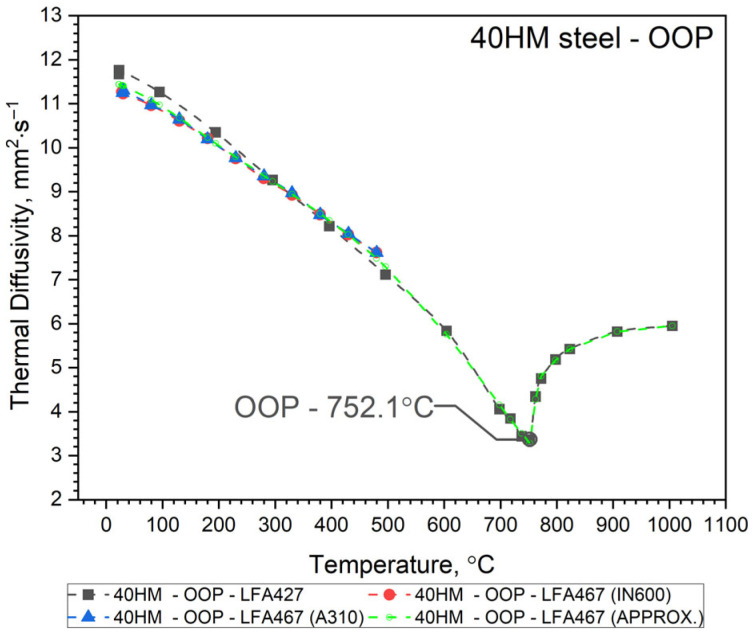
Thermal diffusivity as a function of temperature for 40HM (42CrMo4) steel obtained from LFA 467 and LFA 427: OOP—Out-Of-Plane.

**Figure 5:**
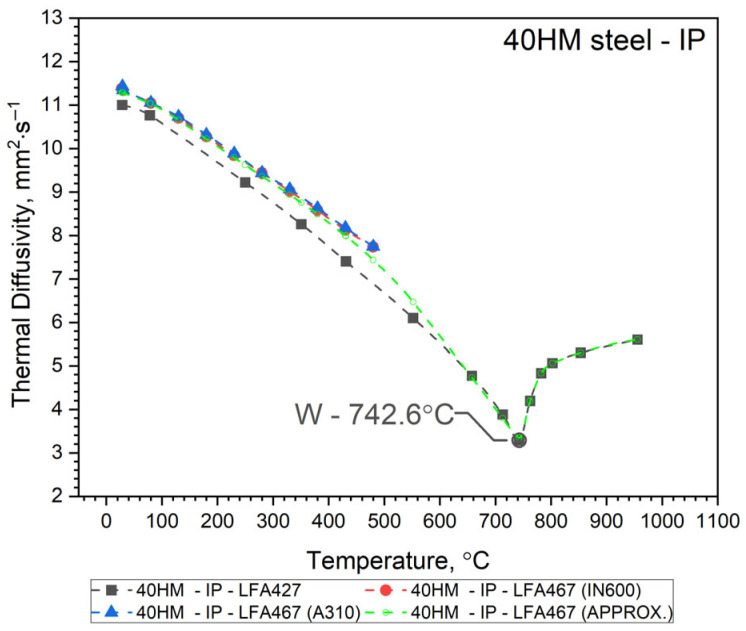
Thermal diffusivity as a function of temperature for 40HM (42CrMo4) steel obtained from LFA 467 and LFA 427: IP—In-Plane.

The thermal diffusivity, *a*(*T*), of 40HM (42CrMo4) steel was measured as a function of temperature from RT to approximately 500 °C using an LFA 467 apparatus (NETZSCH-Gerätebau GmbH, Selb, Germany). These results were compared with measurements taken with an LFA 427 system over an extended range from RT to about 1000 °C. In both cases, thermal diffusivity was determined using the same heat transfer model for the sample, i.e., Cape Lehman + pulse correction, which takes into account the three-dimensional model of the sample and heat losses. Both the LFA 427 and the LFA 467 use the same NETZSCH software (NETZSCH Proteus software version 8). To validate the comparative method for determining the specific heat of 40HM steel using the LFA 467, the thermal diffusivity was measured twice from RT to ~500 °C. The first measurement employed an INCONEL 600 (IN600) reference sample, and the second used an A310 steel reference. Consequently, Figure 3, Figure 4 and Figure 5 each display two distinct *a*(*T*) curves within this lower temperature range. The same figures also present the LFA 427 results for the three sample types from RT to ~1000 °C: the conventionally produced W sample, and the L-PBF samples in out-of-plane and in-plane orientations. Thus, each figure combines the LFA 467 data (RT–500 °C) with the corresponding LFA 427 dataset (RT–1000 °C) for a given sample. The temperature-dependent thermal diffusivity for the 42CrMo4 steel samples (W, OOP, IP) was correlated using polynomial and Shifted Power functions, presented as Equation (1).

Since it turned out that the a(T) characteristics for samples 40HM–W, 40HM–OOP, and 40HM–IP are practically the same—Figure 3, Figure 4 and Figure 5 (green), identical approximation functions were adopted for all three samples. The best fit of the thermal diffusivity results was obtained by the regression method using a second-degree polynomial in the range from the initial temperature $T_{0}$ to peak $T_{1}$ and Shifted Power in the range from the peak $T_{1}$ to the final temperature $T_{2}$—Equation (1).

(1) $$a_{40HM-W,\;OOP,\;IP}=\left\{\begin{array}{c} a_{0}+a_{1}\cdot T+a_{2}\cdot T^{2},\;\mathrm{for}\;T_{0}\leq T\leq \;T_{1}\\ b_{0}{\left(T-b_{1}\right)}^{b_{2}},\;\;\;\;\;\;\;\;\mathrm{for}\;\;\;\;\;\;T_{1}\leq T\leq T_{2} \end{array}\right.$$

The values of coefficients *a_i_* and *b_i_* and temperatures $T_{0},\;T_{1},\;T_{2}$ are given in Table 3, Table 4 and Table 5.

**Figure 6:**
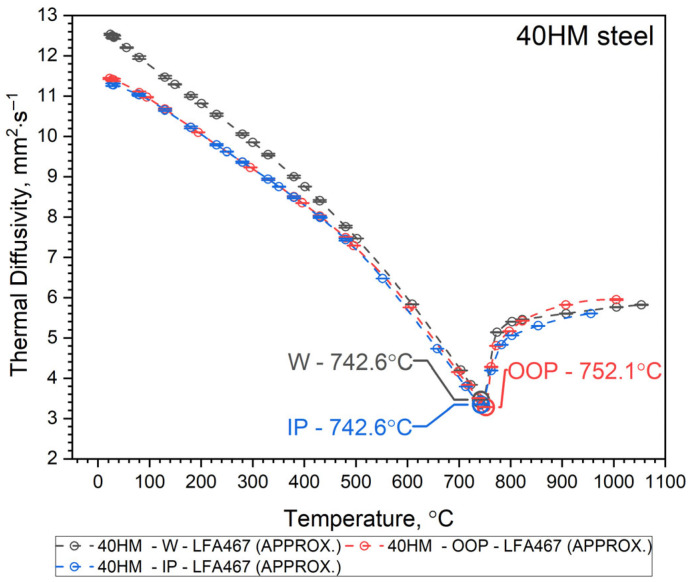
Thermal diffusivity as a function of temperature for 42CrMo4 steel for the samples W, OOP and IP obtained from the approximation Formula (1).

Thermal characteristics of thermal diffusivity *a*(*T*) obtained from the approximation Formula (1) for the W, OOP and IP samples are summarized in Figure 6, and measurement points are shown in Table 6.

### 3.2. Discussion of Thermal Diffusivity Results

Both samples produced by the Laser Powder Bed Fusion (L-PBF) method—specifically the OOP and IP samples—exhibit identical thermal diffusivity characteristics, *a*(*T*), across the investigated temperature range. Minor discrepancies observed between 740 °C and 1100 °C remain within the margin of measurement error. In contrast, sample W, produced via conventional methods, shows a slightly different *a*(*T*) profile from RT to approximately 600 °C. Within this interval, the thermal diffusivity of sample W is higher than that of the L-PBF samples. At room temperature, this difference is approximately 10%, likely due to variations in the concentration of alloying elements and the distinct preparation procedure for sample W relative to the OOP and IP samples (see Table 1). It seems that the porosity of the OOP and IP samples is the main reason for the lower thermal diffusivity of these samples compared to the W sample in the range from RT to 600 °C. All *a*(*T*) characteristics for the 40HM (42CrMo4) medium-carbon steel samples follow a similar trend: a steady decrease in thermal diffusivity from RT toward the Curie temperature, followed by a slight increase. The Curie temperature marking the ferromagnetic-to-paramagnetic transformation occurs at approximately 740 °C for all samples. As the LFA method requires the sample to be thermostated at discrete temperature steps, the recorded Curie temperatures (OOP: 752.1 °C; IP: 742.6 °C; W: 742.6 °C) are considered approximate, reflecting the specific thermostating program and measurement conditions. It should be emphasized that LFA measurements do not allow for the determination of the shrinkage temperature of 40HM steel, as it is a first-order phase transition and is associated with heat input. The thermostating effect prevents this phenomenon from being recorded in LFA measurements.

### 3.3. DSC Investigations

The temperature characteristics of the apparent specific heat capacity were determined using a differential scanning calorimeter DSC 404 F1 Pegasus (NETZSCH, Selb, Germany) in the range of RT—1000 °C. The values of apparent specific heat were calculated using the Cp-ratio method based on the 3-DSC curves (baseline, sapphire line and tested sample line). The test were conducted in a protective atmosphere of argon with 20 mL·min^−1^ flow rate and the heating/cooling rate (HR/CR) was 10 K·min^−1^. In order to obtain stable DSC signals, two evacuations of argon filling the furnace chamber were used along with 15 min isothermal segments after each completed heating/cooling cycle. In addition, temperature and sensitivity calibration of the DSC was performed using five reference materials: indium, bismuth, tin, zinc, and gold.

**Figure 7:**
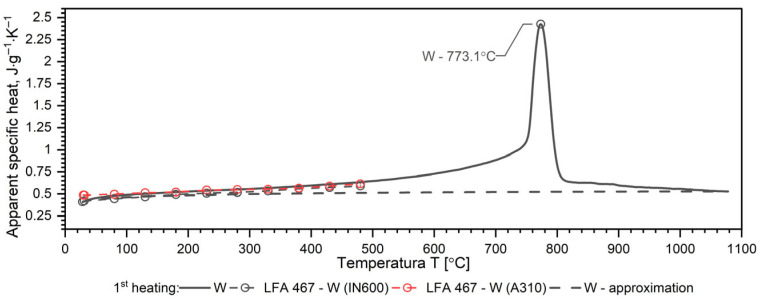
Temperature characteristics of apparent specific heat for the W sample of 40HM (42CrMo4) steel: dashed red line—results obtained from LFA 467, dashed black line—specific heat as a function of temperature *c_p_*(*T*), which can be used for the calculations of thermal conductivity *k*(*T*).

**Figure 8:**
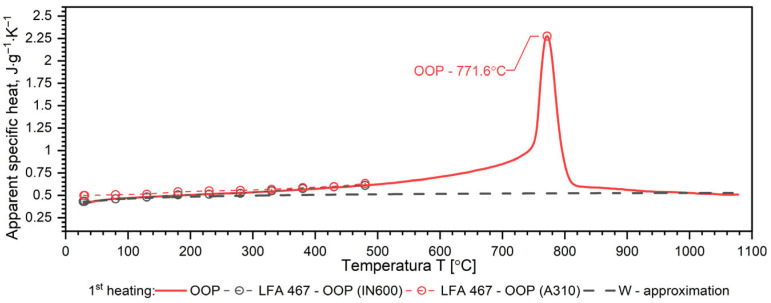
Temperature characteristics of apparent specific heat for the OOP sample of 40HM (42CrMo4) steel: dashed red line—results obtained from LFA 467, dashed black line—specific heat as a function of temperature *c_p_*(*T*), which can be used for the calculations of thermal conductivity *k*(*T*).

**Figure 9:**
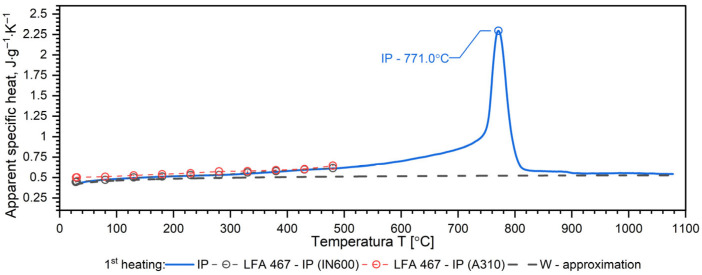
Temperature characteristics of apparent specific heat for the IP sample of 40HM (42CrMo4) steel: dashed red line—results obtained from LFA 467, dashed black line—specific heat as a function of temperature *c_p_*(*T*), which can be used for the calculations of thermal conductivity *k*(*T*).

**Figure 10:**
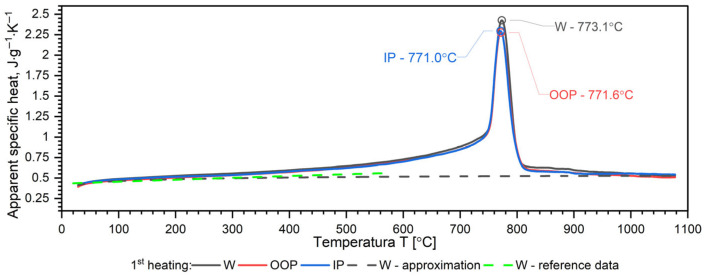
Temperature characteristics of apparent specific heat for the W, OOP and IP samples of 40HM (42CrMo4) steel: dashed black line—specific heat as a function of temperature *c_p_*(*T*), which can be used for the calculations of thermal conductivity *k*(*T*). Green line based on data [38].

A separate problem connected with DSC measurements is the method of calculating the specific heat as a function of temperature in order to obtain input data for simulations of heat transfer. Thermal diffusivity *a*, thermal conductivity *k*, specific heat *c_p_* and density *ρ* are related to the expression *a* = *k*/(*ρ*·*c_p_*). Each of these thermophysical parameters can be determined on separate measuring setups. The phase transformation is visible in each thermophysical parameter. Thus, when calculating the thermal conductivity *k* in the phase transition region from formula *k* = *a·ρ·c_p_*, this effect is taken into account both in thermal diffusivity and in specific heat. This means that the phase change effect and the associated enthalpy are taken into account twice. To avoid this, the phase transition effect is taken into account in the thermal conductivity characteristics, while the thermal characteristics of the specific heat are introduced into the equation $k(T)=\rho \left(T\right)\cdot a(T)\cdot c_{p}(T)$ in the form of an approximation of the thermal characteristics of the apparent specific heat obtained from DSC measurements. The method of approximation of specific heat using apparent specific heat is shown in Figure 7, Figure 8, Figure 9 and Figure 10 and explained in [34,35,36,37].

The results of the apparent specific heat investigations for 40HM (42CrMo4) steel over the temperature range of RT–1000 °C are presented in Figure 7, Figure 8 and Figure 9. These data were obtained from Differential Scanning Calorimetry (DSC) measurements for the W, OOP, and IP samples, with one measurement cycle performed per sample. Figure 7, Figure 8 and Figure 9 also display the specific heat determined via the comparative method using the LFA 467 within the RT–500 °C range. The *c_p_*(*T*) characteristics in this interval were derived using INCONEL 600 and A310 reference samples. For both reference materials, the resulting *c_p_*(*T*) dependencies coincide, further validating the accuracy of the comparative procedure. Additionally, the dashed line across the RT–1000 °C range represents the specific heat function, *c_p_*(*T*), which is utilized for calculating thermal conductivity, *k*(*T*).

The results of apparent specific heat measurements as a function of temperature for samples W, OOP and IP of 40HM (42CrMo4) steel practically coincide—Figure 10. The phase transition peak temperatures for samples OOP: 771.6 °C and IP: 771.0 °C differ little. The peak temperature of sample W is 773.1 °C, which is approximately 3 °C higher than the samples produced using the L-PBF technique, which results from a slightly different percentage of alloying elements—Figure 10. In quantitative terms, the peak temperature position corresponds to a mean temperature of 772.05 °C and a deviation of 1.05 °C.

Since the *c_p_*(*T*) characteristics for the W, OOP and IP samples overlap (dashed black lines), a single correlation formula was proposed for the tested temperature range, from RT to 1000 °C. The proposed formula has the following form:

(2) $$c_{p,40HM-W,OOP,IP}=a_{0}+\;a_{1}\cdot T\;+\;a_{2}\cdot T^{2}+\;a_{3}\cdot T^{-\frac{1}{3}}$$

The values of coefficients *a_i_* are given in Table 7.

### 3.4. Discussion of DSC Results

Both samples produced via the L-PBF method (OOP and IP) exhibit identical apparent specific heat characteristics across the investigated temperature range. In contrast, sample W, produced by conventional methods, shows a slightly higher DSC peak temperature, which is attributed to variations in the concentration of alloying elements. In medium-carbon steels with a composition similar to 40HM, two distinct phenomena typically occur: a magnetic phase transition associated with the Curie temperature and a structural transition related to material shrinkage [34,39,40]. For the 40HM steel studied here, these transitions overlap due to the relatively low chromium content. In steels where chromium exceeds 1%, the Curie temperature remains stable while the shrinkage temperature increases, resulting in two distinct peaks in DSC measurements [34,35]. The literature data *c_p_*(*T*) in the range from RT to about 600 °C coincide with the measured results.

### 3.5. Thermal Expansion Results

A NETZSCH pushrod dilatometer (DIL 402C) was used to measure the thermal expansion of the steel from RT to 1100 °C. Calibration was performed using a standard sapphire reference (diameter 6 mm, length 25 mm). Both the sample and the reference material were subjected to the same thermal cycle: an initial standby at 25 °C, heating to 1100 °C at a rate of 2 K/min, an isothermal hold at 1100 °C for 15 min, followed by cooling to 25 °C at 2 K/min and a final 15 min isothermal hold. The pushrod contact force was set to 15 cN, and the measurement chamber was purged with argon at a flow rate of 50 mL/min. The relative thermal expansion, $\varepsilon \left(T\right)=∆L/L_{0}$, and the coefficient of linear thermal expansion ${CLTE}^{*}\left(T\right)$ were recorded. ${CLTE}^{*}\left(T\right)$ was given in relation to the initial length of the sample *L*(*T_0_*), as follows [41]:

(3) $${CLTE}^{*}\left(T\right)=\frac{1}{L\left(T_{0}\right)}\cdot \frac{dL\left(T\right)}{dT}=\frac{1}{L_{0}}\cdot \frac{dL\left(T\right)}{dT}$$

Taking into account the expansivity of the sample, $\varepsilon \left(T\right)$, its density *ρ(T)* was calculated according to Formula (4):

(4) $$\rho \left(T\right)=\frac{\rho_{0}}{{\left[1+\varepsilon \left(T\right)\right]}^{3}}$$

**Figure 11:**
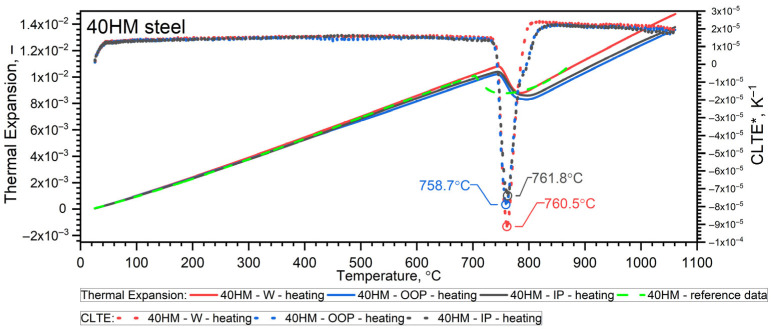
Measurement results $\varepsilon \left(T\right)$ and CLTE*(T) for the W, OOP and IP samples of 42CrMo4 steel in the heating cycle [31]. Green line based on data [42].

**Figure 12:**
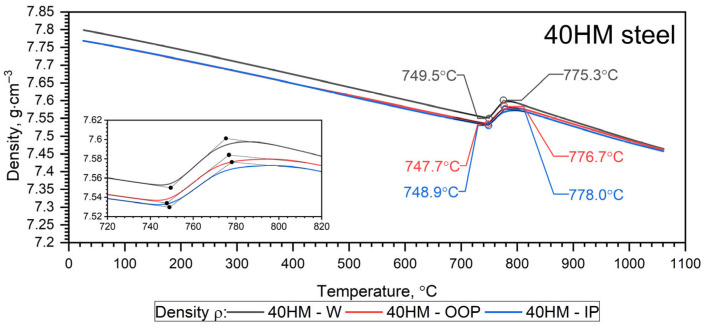
Measurement results density $\rho \left(T\right)$ for the W, OOP and IP samples of 40HM (42CrMo4) steel in the heating cycle [31].

The temperature-dependent density ρ(T) calculated from dilatometric data represents the intrinsic (true) density of the solid matrix, derived from thermal expansion under the assumption of a continuous material. The independently measured porosity of the L-PBF samples (0.3% from metallography and 1.07% from CT) is treated separately as a structural characteristic. Given the low porosity level, the difference between true density and effective bulk density is considered negligible within the experimental resolution of the present study. The results of dilatometric tests of 40HM (AISI 4140) steel in the heating cycles are shown in Figure 11 and Figure 12 and summarized in Table 8 [31]. The density $\rho_{0}$ of the W sample was equal to 7.80 g·cm^−3^, while for the L-PBF samples (OOP and IP), it was 7.77 g·cm^−3^.

Since it turned out that the characteristics $\rho \left(T\right)$ for the 40HM—OOP and 40HM—IP samples are practically the same—Figure 12. The results for both cases are presented in Table 8.

**Figure 13:**
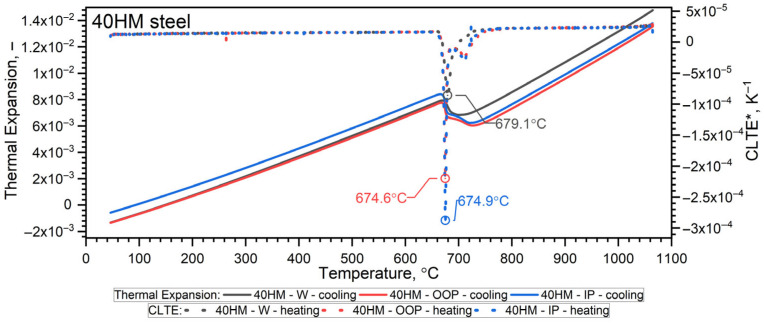
Thermal expansion results $\varepsilon \left(T\right)$ for tested 40HM steel in the cooling cycle [31].

**Figure 14:**
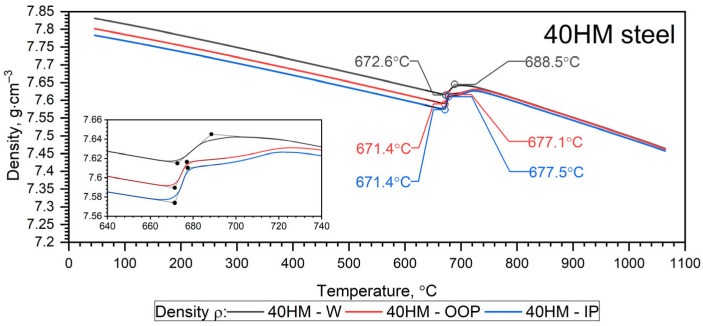
Density results $\rho \left(T\right)$ for tested 40HM steel in the cooling cycle [31].

The results of dilatometric tests of 40HM (AISI 4140) steel in the cooling cycles are shown in Figure 13 and Figure 14.

### 3.6. Discussion of DIL Results

#### 3.6.1. Heating Cycle

Both samples produced via the L-PBF method (OOP and IP) exhibit identical characteristics for thermal expansion ε(T), the coefficient of linear thermal expansion ${CLTE}^{*}\left(T\right)$, and density ρ(T) across the investigated temperature range. Sample W, produced by conventional methods, exhibits characteristics that differ only slightly, primarily between the shrinkage temperature (760 °C) and approximately 1000 °C. Furthermore, dilatometric analysis confirms that the shrinkage temperature of 40HM steel coincides with the Curie temperature identified in the thermal diffusivity measurements. Previous research by the authors indicates that the shrinkage temperature in medium-carbon steels with compositions similar to 40HM is highly dependent on chromium content [34,35]. For instance, in steels with a chromium content of approximately 5%, the shrinkage temperature can exceed the Curie temperature by as much as 100 °C [31,36]. The literature data ε(T) in the range from RT to about 900 °C coincide with the measured results $\varepsilon_{40HM-W,OOP,IP}(T)$. The 40 °C lower shrinkage temperature in the reference steel is most likely due to the slightly lower chromium content (from 0.8% to 1.1%) [38]. It should be emphasized that repeatedly exceeding the shrinkage temperature during operation of the devices made of 40HM steel results in the formation of cracks and degradation of the steel surface.

#### 3.6.2. Cooling Cycle

Minor disturbances in the thermal expansion $\varepsilon \left(T\right)$ during the phase transformation associated with shrinkage in the L-PBF samples are attributed to the inherent heat-treatment effects of the additive manufacturing process (Figure 13 and Figure 14). Because each measurement cycle lasted 18 h, only one cycle was performed per sample; consequently, these thermal expansion data should be regarded as representative of individual specimens.

**Figure 15:**
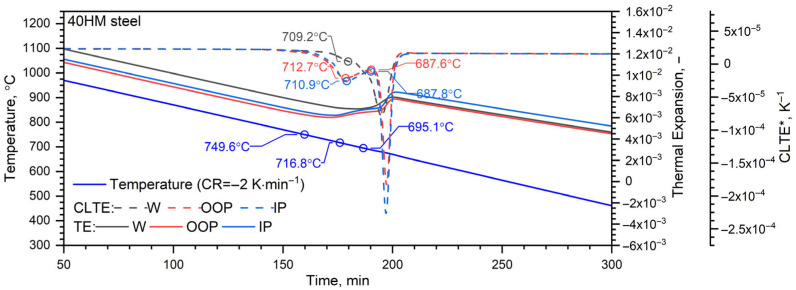
Thermal expansion and temperature of samples manufactured using L-PBF technology (OOP, IP) and sample W (RAW) during cooling in function of time for tested 40HM steel [31].

**Figure 16:**
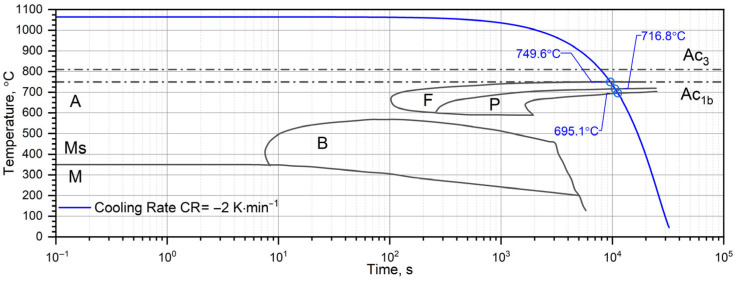
Continuous Cooling Transformation (CCT) chart with cooling program of sample for tested 40HM steel; description: A—austenite, B—bainite, F—ferrite, P—perlite, M—martensite, Ms—start temperature of martensite transformation, Ac_3_—start and Ac_1b_—end temperature of austenite transformation [45].

Figure 15 illustrates the cooling curves (temperature vs. time) alongside the thermal expansion $\varepsilon \left(T\right)$ and ${CLTE}^{*}\left(T\right)$, as functions of time for both the L-PBF and conventional (W) samples. Figure 16 provides a literature-based Continuous Cooling Transformation (CCT) diagram overlaid with the recorded cooling profiles. At a cooling rate of 2 K/min, 40HM steel first undergoes a ferritic transformation at 749.6 °C, followed by a pearlitic transformation between 716.8 °C and 695.1 °C.

CLTE analysis reveals peaks corresponding to these transformations. Sample W exhibits a primary peak at 679.1 °C and a subtle inflection at 709.2 °C. In contrast, the L-PBF samples are characterized by distinct peaks at 712.7 °C (OOP) and 710.9 °C (IP)—marking the onset of pearlite formation—and subsequent maxima at 687.6 °C (OOP) and 687.8 °C (IP), representing the completion of the process. Discrepancies relative to the literature data may stem from variations in sample preparation that influence transformation kinetics or differences in alloying element concentrations [43,44]. At the cooling rate employed, no bainitic transformation was observed.

For both L-PBF specimens and the W specimen, the thermal-expansion response $\varepsilon \left(T\right)$ and the coefficient of linear thermal expansion ${CLTE}^{*}\left(T\right)$ exhibit essentially the same behavior.

## 4. Conclusions

It should be noted that the chemical compositions of the conventionally produced and L-PBF-manufactured materials are not strictly identical (Table 1), and chemical composition should therefore be regarded as a potential confounding factor when interpreting the observed differences in thermophysical properties. However, the compositional variations are minor and remain within the standard specification range for 42CrMo4 steel; consequently, their influence on the measured bulk thermophysical properties is expected to be secondary compared to the effects of temperature and phase transformations. The present comparison is intended to assess the thermophysical properties of 42CrMo4 steel in its characteristic final states resulting from conventional manufacturing and L-PBF processing, rather than to strictly isolate the effect of the manufacturing route from the final metallurgical condition. It should be noted that the present conclusions are valid for the investigated materials in their characteristic final metallurgical states and at the macroscopic measurement scale employed. The potential influence of microstructural features at smaller spatial scales cannot be excluded and would require dedicated high-resolution investigations.

The thermal characteristics—specifically thermal diffusivity *a*(*T*), apparent specific heat from DSC investigations, specific heat *c_p_*(*T*), thermal expansion $\varepsilon \left(T\right)$, coefficient of linear thermal expansion ${CLTE}^{*}\left(T\right)$, and density $\rho \left(T\right)$ are virtually identical for the L-PBF and conventional (W) 40HM steel samples. Minor discrepancies arise from variations in alloying element concentrations and the distinct preparation procedures inherent to the L-PBF technique versus conventional manufacturing, both of which influence transformation kinetics. The greatest differences occur in the thermal characteristics of thermal diffusivity, which is related to the porosity of samples made using the L-PBF technique. Porosity does not affect specific heat, as this is a property of the steel itself (iron and alloying elements). Porosity also has no effect on thermal expansion, as this depends on vibrations in the steel’s crystal lattice.

**Figure 17:**
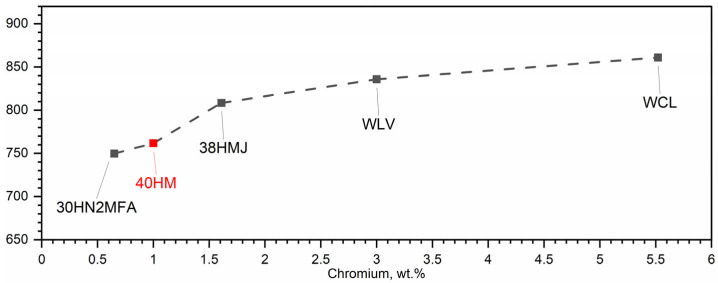
Influence of chromium content on shrinkage temperature of selected barrel steels, i.e., 30HN2MFA, 38HMJ, WLV, WCL and 40HM (AISI 4140) [31,34].

In 40HM steel, the transformation associated with shrinkage coincides with the ferromagnetic-to-paramagnetic transition at the Curie temperature. This overlap of phase transformations is attributed to the low chromium content (approximately 1% by weight) of 40HM steel. Having investigated medium-carbon steels for several years, the authors conclude that chromium content is the primary driver of the shrinkage temperature: increased chromium concentrations yield higher shrinkage temperatures [34,36,37]. Figure 17 summarizes this trend for selected steels—30HN2MFA, 38HMJ (41CrAlMo7-10), WLV (32CrMoV12-28), WCL (X37CrMoV5-1), and 40HM—measured during heating [34,35]. As a ferrite stabilizer, chromium promotes ferrite formation and consequently elevates the austenite-to-ferrite transformation temperature during thermal cycling. Accordingly, chromium additions shift the shrinkage temperature upward. In steels where chromium exceeds 1%, the shrinkage temperature no longer coincides with the Curie temperature; for a chromium content of 5%, the shrinkage temperature exceeds the Curie temperature by as much as 100 °C [34,36,37].

## Figures and Tables

**Table 1:** Chemical composition of 40HM steel (AISI 4140, 42CrMo4, 1.7225) [32,33].

		C	P	S	Mo	Mn	Cr	Si
Weight (%)	Powder	0.39	0.006	0.007	0.2	0.9	1.0	0.3
	Wrought	0.42	0.01	0.004	0.15	0.83	1.06	0.23

**Table 2:** Samples for testing the thermophysical properties of 40HM steel: diameter—*d*, thickness (length in case of DIL)—*l*.

40HM (42CrMo4) Steel Sample		LFA	DSC	DIL
W	*d*, mm	12.55	5.53	5.53
	*l*, mm	2.07	1.58	24.75
OOP	*d*, mm	12.60	5.53	5.53
	*l*, mm	2.10	1.58	24.95
IP	*d*, mm	12.55	5.53	5.53
	*l*, mm	2.04	1.58	25.15

Samples for DSC investigations were placed in the platinum crucible with the platinum lid (volume of Pt crucible: 85 µL). The weight of the 42CrMo4 steel samples were W—294.90 mg, OOP—282.64 mg, IP—285.00 mg.

**Table 3:** Coefficients for calculating thermal diffusivity of 40HM-W samples on Equation (1).

24.2 °C≤T≤742.6 °C		742.6 °C<T≤1053.7 °C	

$a_{0}[{mm}^{2}s^{-1}]$	$1.25911\cdot 10^{1}$	$b_{0}[{mm}^{2}s^{-1}{℃}^{-b_{2}}]$	$4.35193\cdot 10^{0}$
$a_{1}[{mm}^{2}s^{-1}{℃}^{-1}]$	$-6.90269\cdot 10^{-3}$	$b_{1}[℃]$	$7.42586\cdot 10^{2}$
$a_{2}[{mm}^{2}s^{-1}{℃}^{-2}]$	$-7.05854\cdot 10^{-6}$	$b_{2}[-]$	$5.04687\cdot 10^{-2}$
$R^{2}=0.9928$		$R^{2}=0.9986$	

**Table 4:** Coefficients for calculating thermal diffusivity of 40HM-OOP samples on Equation (1).

22.5 °C≤T≤752.1 °C		752.1 °C<T≤1005.0 °C	

$a_{0}[{mm}^{2}s^{-1}]$	$1.15018\cdot 10^{1}$	$b_{0}[{mm}^{2}s^{-1}{℃}^{-b_{2}}]$	$3.52853\cdot 10^{0}$
$a_{1}[{mm}^{2}s^{-1}{℃}^{-1}]$	$-5.05429\cdot 10^{-3}$	$b_{1}[℃]$	$7.51485\cdot 10^{2}$
$a_{2}[{mm}^{2}s^{-1}{℃}^{-2}]$	$-7.68886\cdot 10^{-6}$	$b_{2}[-]$	$9.74209\cdot 10^{-2}$
$R^{2}=0.9954$		$R^{2}=0.9938$	

**Table 5:** Coefficients for calculating thermal diffusivity of 40HM-IP samples on Equation (1).

2 °C≤T≤752.1 °C		752.1 °C<T≤1005.0 °C	

$a_{0}[{mm}^{2}s^{-1}]$	$1.13879\cdot 10^{1}$	$b_{0}[{mm}^{2}s^{-1}{℃}^{-b_{2}}]$	$3.08708\cdot 10^{0}$
$a_{1}[{mm}^{2}s^{-1}{℃}^{-1}]$	$-4.48815\cdot 10^{-3}$	$b_{1}[℃]$	$7.40933\cdot 10^{2}$
$a_{2}[{mm}^{2}s^{-1}{℃}^{-2}]$	$-8.43401\cdot 10^{-6}$	$b_{2}[-]$	$1.13272\cdot 10^{-2}$
$R^{2}=0.9885$		$R^{2}=0.9801$	

**Table 6:** Thermal diffusivity measurement results for samples W and L-LBF along with measurement uncertainties at each temperature point.

40HM—W		40HM—OOP		40HM—IP	
*T* [°C]	$a\;[{mm}^{2}s^{-1}]$	*T* [°C]	$a\;[{mm}^{2}s^{-1}]$	*T* [°C]	$a\;[{mm}^{2}s^{-1}]$
24.2	12.54 ± 0.023	22.5	11.44 ± 0.019	28.0	11.29 ± 0.032
28.0	12.49 ± 0.041	22.7	11.44 ± 0.018	28.7	11.28 ± 0.035
29.5	12.48 ± 0.054	28.0	11.42 ± 0.031	28.8	11.28 ± 0.033
30.9	12.46 ± 0.039	29.2	11.41 ± 0.029	30.0	11.28 ± 0.032
31.1	12.46 ± 0.042	30.4	11.40 ± 0.031	30.2	11.28 ± 0.032
55.5	12.2 ± 0.010	30.6	11.40 ± 0.030	78.4	11.04 ± 0.015
80.0	11.96 ± 0.037	80.0	11.09 ± 0.027	80.0	11.03 ± 0.028
130.0	11.47 ± 0.034	94.6	10.97 ± 0.010	130.0	10.65 ± 0.027
149.2	11.29 ± 0.007	130.0	10.67 ± 0.025	180.0	10.23 ± 0.026
180.0	11.01 ± 0.031	180.0	10.23 ± 0.023	230.0	9.79 ± 0.023
200.7	10.81 ± 0.005	194.4	10.10 ± 0.005	249.9	9.62 ± 0.007
230.0	10.54 ± 0.033	230.0	9.79 ± 0.021	280.0	9.36 ± 0.022
280.0	10.06 ± 0.03	280.0	9.36 ± 0.019	330.0	8.94 ± 0.023
300.4	9.85 ± 0.004	295.4	9.23 ± 0.003	351.0	8.75 ± 0.006
330.0	9.55 ± 0.026	330.0	8.94 ± 0.021	380.0	8.49 ± 0.024
380.0	9.00 ± 0.030	380.0	8.50 ± 0.0210	430.0	8.00± 0.0240
401.3	8.75 ± 0.003	396.3	8.35 ± 0.002	430.1	8.00 ± 0.0230
430.0	8.41 ± 0.025	430.0	8.03 ± 0.020	431.3	7.99 ± 0.007
480.0	7.76 ± 0.028	480.0	7.48 ± 0.020	480.0	7.44 ± 0.024
501.8	7.46 ± 0.003	495.9	7.29 ± 0.004	551.7	6.47 ± 0.002
609.1	5.83 ± 0.003	604.0	5.76 ± 0.002	657.6	4.73 ± 0.001
703.5	4.19 ± 0.002	698.1	4.16 ± 0.003	712.9	3.79 ± 0.001
722.8	3.84 ± 0.002	717.5	3.83 ± 0.004	742.6	3.34 ± 0.002
742.6	3.46 ± 0.002	737.4	3.51 ± 0.003	762.4	4.19 ± 0.003
773.9	5.14 ± 0.004	752.1	3.28 ± 0.003	782.4	4.83 ± 0.002
802.9	5.40 ± 0.004	762.0	4.28 ± 0.007	802.5	5.06 ± 0.002
822.8	5.45 ± 0.005	772.0	4.80 ± 0.004	853.3	5.30 ± 0.002
907.4	5.60 ± 0.005	797.6	5.17 ± 0.004	955.6	5.60 ± 0.003
1005.2	5.76 ± 0.005	822.9	5.42 ± 0.003		
1053.7	5.82 ± 0.007	907.1	5.82 ± 0.004		
		1005.0	5.95 ± 0.017		

**Table 7:** Coefficients for calculating specific heat capacity for the W, OOP and IP samples of 40HM (42CrMo4) steel on Equation (2).

28.4 °C<T [℃]≤1077.7 °C	

$a_{0}[J\cdot g^{-1}\cdot {℃}^{-1}]$	$5.46648\cdot 10^{-1}$
$a_{1}[J\cdot g^{-1}\cdot {℃}^{-2}]$	$4.63722\cdot 10^{-5}$
$a_{2}[J\cdot g^{-1}\cdot {℃}^{-3}]$	$-2.54780\cdot 10^{-8}$
$a_{3}[J\cdot g^{-1}\cdot {℃}^{-2/3}]$	$-4.09582\cdot 10^{-1}$
$R^{2}=$0.9881	

**Table 8:** Density $\rho \left(T\right)$ for the W, OOP and IP samples of 40HM (42CrMo4) steel in the heating cycle.

W Sample of 40HM Steel: Characteristic $\rho \left(T\right)$		OOP+IP Samples pf 40HM Steel: Approximation from the Both Characteristics $\rho \left(T\right)$
*T* [°C]	$\rho \;[g\cdot cm^{-3}]$	$\rho \;[g\cdot cm^{-3}]$
23	7.800	7.770
50	7.792	7.762
100	7.776	7.747
200	7.744	7.716
400	7.674	7.649
600	7.602	7.580
720	7.560	7.541
730	7.557	7.538
740	7.554	7.535
750	7.555	7.537
760	7.571	7.551
770	7.589	7.567
780	7.597	7.574
790	7.597	7.576
800	7.593	7.576
820	7.583	7.570
850	7.567	7.555
900	7.542	7.531
1000	7.492	7.485
1060	7.465	7.460

## Data Availability

The original contributions presented in this study are included in the article. Further inquiries can be directed to the corresponding author.
